# Effect of Iron Oxide Nanoparticles on the Physical Properties of Medium Density Fiberboard

**DOI:** 10.3390/polym12122911

**Published:** 2020-12-04

**Authors:** Waheed Gul, Hussein Alrobei, Syed Riaz Akbar Shah, Afzal Khan

**Affiliations:** 1 Department of Mechanical Engineering, Institute of Space Technology, Islamabad 44000, Pakistan; waheed.gul@mail.ist.edu.pk; 2 Department of Mechanical Engineering, Prince Sattam Bin Abdulaziz University, Alkharj 11942, Saudi Arabia; 3 Department of Mechatronics Engineering, University of Engineering and Technology, Peshawar 25120, Pakistan; rasayed@uetpeshawar.edu.pk; 4 Department of Mechanical Engineering, University of Engineering and Technology, Peshawar 25120, Pakistan; afzalkhan@uetpeshawar.edu.pk

**Keywords:** natural fiber composite, physical properties, curing temperature, SEM, XRD, EDS, DSC, TGA

## Abstract

This paper investigates the influence of iron oxide (Fe_2_O_3_) nanoparticles on the physical properties of medium density fiberboard (MDF). In this study, three different nano iron oxide loadings, i.e., 0.5, 1.5 and 2.5 wt %, and untreated poplar fibers were used. The iron oxide (Fe_2_O_3_) nanoparticles were initially dispersed into urea formaldehyde resin using a high-vacuum mechanical stirrer before being incorporated into natural fibers. The untreated poplar fibers were wound onto metal frames to produce dry mat layers. Twenty different composite samples were made. All composite samples were tested for physical properties, i.e., thickness swelling, water absorption, moisture content and density in accordance with standards EN-317, ASTM D570, EN-322 and EN-323 respectively. Based on the results, it was found that the incorporation of homogeneously dispersed iron oxide nanoparticles significantly improved thickness swelling (Ts). Moreover, water absorption (WA) improved by up to 49.18 and 34.54%, respectively, at the highest loading of 2.5 wt %. Microstructure was investigated and characterized with scanning electron microscopy (SEM), x-ray diffraction (XRD) and energy dispersive spectroscopy (EDS) and we examined whether iron oxide nanoparticles exhibit good interactions with urea formaldehyde and poplar wood fibers. Heat and mass transfer investigation in the form of differential scanning calorimetry (DSC) and thermogravimetric analysis (TGA) was carried out due to the impact of Fe_2_O_3_ nanoparticles. The curing temperature and thermal stability of the resin were enhanced due to the addition of Fe_2_O_3_ nanoparticles. A one-way ANOVA statistical analysis was established to effectively control the use of Fe_2_O_3_ nanoparticles. Therefore, the presence of iron oxide nanoparticles in an epoxy polymer contributes to a stiffer matrix that, effectively, enhances the capability of improving the physical properties of nano MDF.

## 1. Introduction

**Figure 1:**
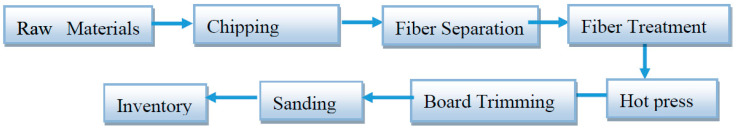
Medium density fiberboard manufacturing process flow.

Wood-based composites are valuable and precious raw materials that have helped mankind establish civilization in the past and present. They are, however, susceptible to bio-deteriorating agents [1]. Therefore, their use is of advantage since they offer a homogeneous structure which is of great importance for many general and specific purposes [2]. Medium density fiberboard (MDF) is a natural fiber composite manufactured in a hydraulic hot press under certain pressure temperature and time [3]. Its applications include furniture industries, loudspeaker boxes, roofing, vapor repulsion, sound proofing, interior cladding for houses and slat walls [4,5,6]. The destiny of medium density fiberboard as per EN-323 standard is 720 ± 20 kg/m^3^. The whole manufacturing process of medium density fiberboard can be seen in Figure 1. The manufacturing process starts withthe preparation of materials in which the poplar wood (raw materials) isreduced into wood chips in a chipper machine [7]. The refined chips are sent to a fiber preparation section where adefiberator converts the refined chips into fibers with the help of steam at 6–8 bar pressure [8].

It must be pointed out that one cannot speak about wood composites without speaking in depth about polymer binders and the adhesives used to hold them together [9]. This plays an important role in the efficient utilization of wood resources and in the development and growth of the forest product industry [10]. The fibers are mixed with 10 wt % urea-formaldehyde resin and further preceded to fiber treatment section where the fibers are dried up to 8–9% moisture content. A prepressing process is performed on the dry fibers and a mat of fibers is formed which is supplied to the hot press. The purpose of hot press is to vaporize the moisture in mat, increase the density and solidify the urea formaldehyde resin under the combined function of temperature, pressure and time, so that MDF with certain physical and mechanical property will be formed after a series of physiochemical reactions [11,12]. The hot pressing process is divided into four stages, i.e., compression of mat to discharge the air, temperature penetration, control of pressurizing to thickness, relief exhaust, and forming. Then it is cooled down in a cooling tower in a board treatment section and sent to a sanding section for removing extra layers and rough surface by means of sanding papers and unwanted cutting of edges. The finished medium density fiberboard is then finally sent to inventory.

The main drawbacks of wood sheet, namely dimensional instability and biological durability, are mainly due to the nature of the cell wall main polymers and in particular due to their high abundance of hydroxyl groups (OH) [13]. At the same time, polycondensation reactions and cross-linking were found to be in a lignin structure [14]. A good cell wall penetration plays an important role in wood modification effects [15].

Nanotechnology is science, engineering, and technology conducted at the nanoscale, which is about 1 to 100 nanometers [16]. Nanotechnology brings an innovative idea of being imported in UF glue and enhancing all characteristics of MDF. Today’s scientists and engineers are finding a wide variety of ways to deliberately make materials at the nanoscale to take advantage of their enhanced properties such as higher strength, lighter weight, increased control of light spectrum, and greater chemical reactivity than their larger-scale counterparts.

Therefore, this study aims to show the effect of iron oxide nanoparticles on the thickness swelling, water absorption, moisture content, and density properties of MDF composites.

Hashim et al. (2005) studied the effect on the fire retardancy and mechanical properties of MDF from recycled corrugated cardboard containing aluminum trioxide (ATH) nanoparticles. The Limited Oxygen Index (LOI)is used as an indicator of fire performance, and the internal restriction increased as ATH loading increased. On the other hand, the swelling of the panel thickness also increased [17]. Lin Qiaojia et al. (2006) investigated the results of nano-SiO_2_ quantitative analysis of pairing agents, sonication methods and nano-SiO_2_/urea formaldehyde resin. Measuring the performance of all three composites, namely plywood, particle board and MDF, by nano-SiO_2_ (1%)/UF resin (F/U molar ratio = 1.2), shows whether the products meet international standard desires [18]. Yong Lei et al. (2007) Prepared, HDPE/Pine composite containing exfoliated clay. When 1% of the soil was accumulated, the MOE and MOR increased by 19.6% and 24.2%, respectively, but then declined to some extent as the soil content increased to 3%. The accumulation of clay enlarged the tensile modules by 11.8% and the exact length by 13%. With the increase in soil content, the external and stereotype modules gradually increased, but the storage and deficit modalities remained at the same level as the clay-clay loading level. Although the impact strength was reduced by 7.5% by an increase of 1% in the soil, it was not further reduced when the soil content increased from 1 to 3% [19]. Hong et al. (2008) showed that a small increase in sodium montmorillonite in UF resin significantly improved its relationship performance. The addition of nanoparticles had a noteworthy effect on the absorption of water and the swelling of the thickness of particle boards bound with UF. Moreover, there was an increase in internal relations. The results showed that sodium montmorillonite accelerated UF resin curing and increased its hardness [20]. Faruk et al. (2008) compared the two methods of adding nanoscale to wood-plastic compounds to enhance the mechanical properties. The first method involved the reinforcement of the HDPE matrix with nanoclay, which was used as a matrix in the preparation of WPCs (melting process). The second method involves the direct incorporation of nanoclay into the HDPE / wood flour mixture during the traditional dry mixing process (direct dry mixing process). The melting process resulted in improved mechanical properties [21]. A. Ashori et al. (2009) described the special effects of nanoclay layered silicate with different concentration level of (0, 2, 4, 6, and 8 wt %) in urea formaldehyde (UF) resin. Physical and mechanical properties of the composites were investigated according to (EN) European Norm standards. The results showed substantial enhancement in mechanical properties, particularly in bending strength and I.B strength of the composites as nanoclay was added with concentration from 2 to 6 wt %. The composite with concentration level 6 wt % indicates the best mechanical properties. There was an understandable tendency that both thickness swelling (after 2 and 24 h) reduced with accumulation of nanoclay and hot press temperature [22]. Z. sheijani R. et al. (2011) studied the effect of nano-silver on physical and mechanical properties of particleboard (PB) finished on commercial scale. Nano-silver suspension was accumulated to the fibers at two levels of 100 and 150 mL/kg dry weight. The hot pressing parameters were kept constant, except for the time parameter. The consequences obtained showed a 10.9% and 10.1% reduction of hot pressing time. Also, these two concentrations of nano-silver enhanced physical and mechanical properties. It can be accomplished that thermal characteristics of nano-silver particles in the mat can be used to decrease the pressing time [23]. In a separate study, Xian, D et al. (2013) highlighted the improvement of particleboard properties with nanoclay of concentration level 2% to melamine formaldehyde resin. A significant improvement in both internal bonding and swelling in thickness were investigated. Further, the swelling in thickness was reduced to an optimum value by the addition of 6% nanoclay [24]. Taghiyari, H. R et al. (2014) studied the enlightening effect of a raise in the thermal conductivity due to nano-wollastonite (NW) on the physical and mechanical properties of MDF. Nanowollastonite was added at different concentration levels of 2, 4, 6, and 8 g/kg, based on the dry weight of wood-specimens. The findings show that NW considerably (*p* < 0.05) augmented thermal conductivity. The improved thermal conductivity occasioned in an improved curing of UF resin; as a result, mechanical properties were enhanced expressively. In addition, the configuration of bonds involving wood fibers and wollastonite accomplished to stimulating the MDF. It was determined that a NW concentration of 2 g/kg did not appreciably improve the whole properties and therefore cannot be suggested to the industry. Since the properties of NW-6 and NW-8 were drastically comparable, a NW-content of 6 g/kg can be suggested to the industry to radically (*p* < 0.05) advance the properties of MDF sheets [25]. Candan, Z et al. (2015), developed a novel preferential to concert properties acquired through non-technology. The main objective of this research was to develop nanomaterial reinforced particle boards with better physical and mechanical properties. In this study, a UF nano filer with nano SiO_2_, nano Al_2_O_3_, and nano ZnO was used at three concentration levels of 0, 1, and 3%. Tests for rupture modules, elasticity modules, bonding strength, and screw return strength tests were performed to assess the mechanical properties of particle board alloys while the physical properties were identified as density, thickness, swelling, water absorption, and balance moisture content. The results obtained in this work revealed that the nanomaterial support approach has made the physical and mechanical properties of the particle board admirable. The results show that the screw return resistance of the compound increased by rupture modules, elasticity modules, bonding strength, and all the nanometers used in this learning, except for 3% nano ZnO. It was also determined that the use of 1% nano-SO_2_ or 1% nano-Al_2_O_3_ in the particle board yielded the best results in bonding strength and screw withdrawal resistance [26].

The special effects of wollostonite fibers on the physical and mechanical properties of medium-density fiber boards were investigated by Taghiyari et al. (2016). Around 30% of the wallostonite fibers were less than 100 nm while the rest were less than 1 μm. Wollostonite fibers have significantly improved most physical and mechanical properties, while the addition of camel hooks has had a more variable effect on panel properties. A 10% camel hook can be added to the panel without any side effects. The combination of 10 camel forks and 5 wollostonite fibers produced panels with excellent properties [27]. The study conducted by N. Ismita et al. (2017) explore the effects of adding a Na+ (nanoclay) to UF resin on the physical and mechanical properties of particle boards. Cloisite Na+ was introduced at three different concentration levels of 2, 4, and 6% in UF. Physical properties (i.e., density, water absorption (WA), thickness swelling (TS)) and mechanical properties (i.e., modulus of rupture (MOR), modulus of elasticity (MOE) and internal bond strength (IB)) were deliberated to evaluate the concert of the composites. Considerable increase was observed for TS, MOR, and MOE properties. More particularly, in composites bonded with UF resin and 6% concentration of nanoclay, 34 and 65% improvements were achieved in MOR and MOE respectively, equating to the control composites [28]. Yipeng Chen et al. (2018) developed a hasty and lime procedure for the amalgamation of lignocelluloses-based composites with better mechanical properties. Specimens were shaped by multi opening hot-pressed technique using diverse concentrations of calcium carbonate (CaCO_3_) and poly (methyl methacrylate) particles-filled nan lignocelluloses. MOR, MOE, and dimensional stability and thermogravametic analysis of the established lignocelluloses centered composites were carried out. In observation of the experimental findings, it is clear that the nano composites have superior mechanical and dimensional stability, and thermal properties, which improved as the filler concentration increased [29]. The use of wood base panels in humid environments, in general, offers low stability due to contact with water. Studies were conducted using resin with zinc oxide (ZnO) nanoparticles to increase stability and reduce the invasion of fungi. The purpose of this work is to develop a medium density fiber board of 0.5 and ZnO nanoparticles with urea-formaldehyde resin and melamine formaldehyde for the evaluation of physical properties. All treatments were classified as medium density with values between 550 and 800 kg/m^3^. No differences were found between the two commercial adhesives used. The nanoparticles increased by 1.0% resulting in lower density panels, higher moisture content, and higher values of swelling in thickness after 24 h of immersion in water. These results are explained by the lower compression of the board due to the faster treatment of the adhesive using a higher percentage of ZnO nanoparticles. The best treatment for nanocomposite panel was with melamine-formaldehyde resin and 0.5% nanoparticles [30]. Alabduljabbar, H et al. (2020) explored the effect of Al_2_O_3_ nanoparticles on the physical and mechanical properties of nano-MDF. The Al_2_O_3_ nanoparticles were introduced at 0, 1.5, 3, and 4.5% in the urea-formaldehyde resin and the final internal bonding, modulus of elasticity, modulus of rupture, thickness swelling and water absorption properties were enhanced up to 16.4, 31, 22.12, 40.15, and 37.53%, respectively [31].

A brief literature review of nanoparticle addition in UF glue to enhance physical and mechanical characteristics of MDF is given in Table 1.

The characteristics of nanoparticles-based MDF are characterized using a literature review of various types of nanoparticles. Various nanoparticles are plotted versus all the physical and mechanical properties. Physical properties of MDF includedensity, water absorption (WA) and thickness swelling (TS) while mechanical properties include modulus of elasticity (MOE) (MPa), modulus of rupture (MOR) (MPa), and internal bond (I.B) (MPa).

Internal bond (I.B) is plotted versus various nanoparticles used in MDF. Regarding the manufactured MDF samples from Hashim et al. (2005) [17], Yong Lei et al. (2007) [19], Taghiyari et al. (2014) [25], and Yipeng Chen et al. (2018) [29] for Aluminum trihydroxide(ATH), Nanoclay, nano-wollastonite and CaCO_3_/PMMA, the experimental values of thephysical properties, i.e.,water absorption, fluctuated between 50 and 55%. For nano-reinforced and wollastonite, the value of water absorption wascalculated as 93 and 78%. These values are not feasible for standard MDF, and ultimately the composites produced will be weak. Faruk et al. (2008) [20] and Alabduljabbar et al. (2020) [31] investigated the safest value of WA (13% and 13.73%) for nanoclay-based quality MDF.

## 2. Materials and Methods

### 2.1. Materials

Urea-formaldehyde resin, iron oxide (Fe_2_O_3_) nanoparticles and poplar wood fibers are used as raw materials for manufacturing nano medium density fiberboard. These raw materials are explained in the subgroup below.

#### 2.1.1. Urea-Formaldehyde Resin

The leading manufacturing group Wah Nobel group, Wah Cantt., Pakistan, delivered urea-formaldehyde glue with the following specifications as presented in Table 2.

#### 2.1.2. Iron Oxide (Fe_2_O_3_) Nanoparticles

Iron oxide (Fe_2_O_3_) nanoparticles with size 90–150 nm were purchased from YIPIN Pigments GmbH Company, Hamburg, Germany. The choice of iron oxide (Fe_2_O_3_) nanoparticles was madeowing to its antibacterial effect, anti-cancer, magnetic behavior, and semiconductor properties [25].

#### 2.1.3. Poplar Wood Fibers

Poplar wood fibers were received from Frontier Green Wood Industries (Pvt.) Ltd., Peshawar, Pakistan. The length of the fibers ranges from 0.4 to 1 mm.

### 2.2. Preparation of UF-Fe_2_O_3_ Nanofiller

The Fe_2_O_3_-UF nanofluid was primed in the Institute of Space Technology, Islamabad, Pakistan materials Science Work room according to the configurations specified in Table 3.

**Figure 2:**
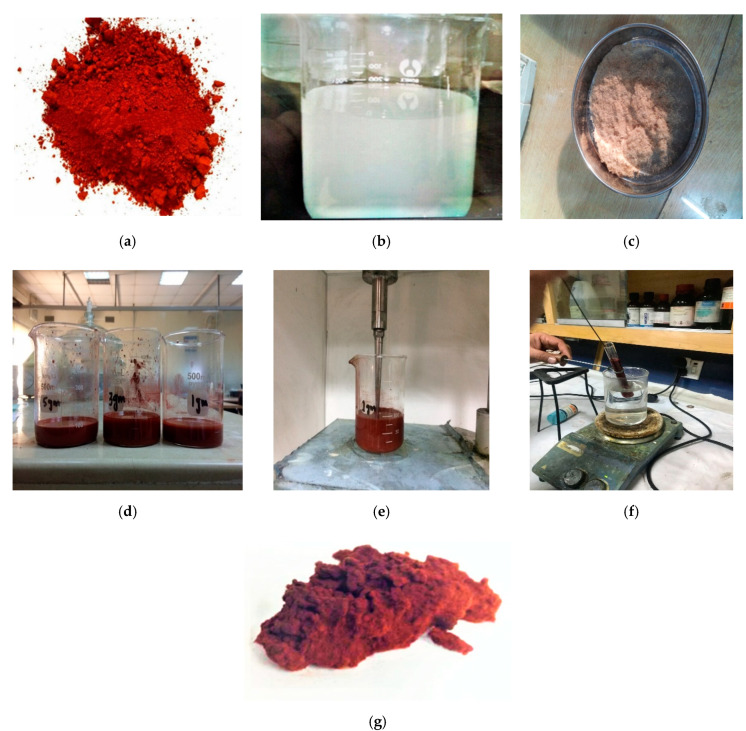
(**a**) Iron Oxide (Fe_2_O_3_) nanoparticles, (**b**) urea formaldehyde glue, (**c**) medium density fiberboard (MDF) fibers without nanofillers, (**d**) preparation of Fe_2_O_3_ and urea-formaldehyde nanofillers, (**e**) sonication of nanofillers, (**f**) curing of nanofillers, (**g**) natural fibers mixed with (Fe_2_O_3_) nanoparticles.

The nanofluids were blended by weighing 200 g of urea-formaldehyde resin and 0, 1.3 and 5 g of iron oxide (Fe_2_O_3_) nanoparticles of dry weight of fibers. The sonication of the nanofluids was carried out by means of Ultrasonic Processor UP 400S of Hielscher Ultrasound Technology Company, USA for 30 min. The samples were signified by Fe_0_, Fe_1_, Fe_2_ and Fe_3_ rendering to the meditation of Fe_2_O_3_. The iron oxide (Fe_2_O_3_) nanoparticles, urea-formaldehyde resin, poplar wood fibers, nanofillers, sonication and curing of the fillers can be seen in Figure 2.

### 2.3. Nano Natural Composite Design

The nano natural composite testers were manufactured in hot plates with sizes 450 × 450 × 15 mm and densities ranging from 700 kg/m^3^ to 750 kg/m^3^. The iron oxide (Fe_2_O_3_)-UF nanofluids were well mixed with poplar wood fibers in a rotary drum fiber mixer with a nozzle. A single opening hot press by (BURKLE, Bohemia, NY, USA) operated hydraulically was used for manufacturing nano MDF samples. The hot pressing process parameters, i.e., pressure 162 bar and temperature 168 °C, were kept constant for all testers. The whole press cycle was 4.2 min and the manufactured samples were treated in a cooling tower for 3 days.

### 2.4. Scanning Electron Microscopy (SEM) of Iron Oxide (Fe_2_O_3_) Nanoparticles

**Figure 3:**
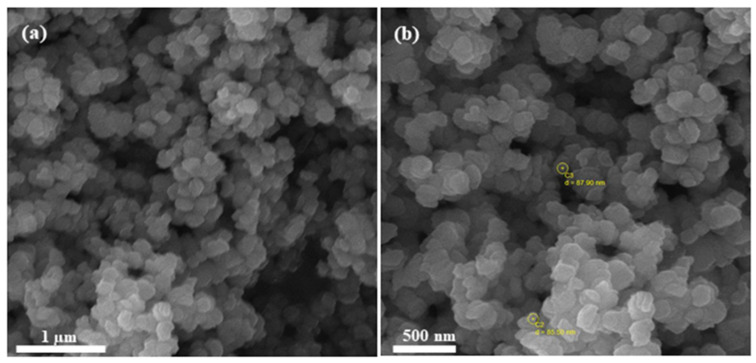
Scanning electron microscopy metaphors of Iron Oxide Fe_2_O_3_ at (**a**) 25,000×, (**b**) 50,000×.

Scanning Electron Microscopy was carried out in the SEM Laboratory of Institute of Space Technology, Islamabad, Pakistan. Before being subjected to SEM, a sample of iron oxide (Fe_2_O_3_) nanopowder was prepared in the laboratory and coated with gold by means of Safematic CCU-010 Gold/Carbon Sputter, (Labtech International Ltd., Heathfield, UK). SEM was accomplished with MIRA3 (TESCAN, Brno, Czech Republic) at intensifications of 25,000× and 25,000× with an extreme working voltage of 20 kV, as given in Figure 3.

### 2.5. Energy Dispersive X-ray Spectroscopy (EDS) Analysis of Iron Oxide (Fe_2_O_3_) Nanoparticles

Energy dispersive X-ray spectroscopy was implemented with MIRA3 (TESCAN, Brno, Czech Republic) at magnifications of 25,000× and 50,000× with an extreme working voltage of 20 kV on the region plotting of SEM images. This depiction was approved to check the existence of Fe_2_O_3_ nanoparticles in the UF glue.

### 2.6. X-ray Diffraction Analysis of Fe_2_O_3_ Nanoparticles

**Figure 4:**
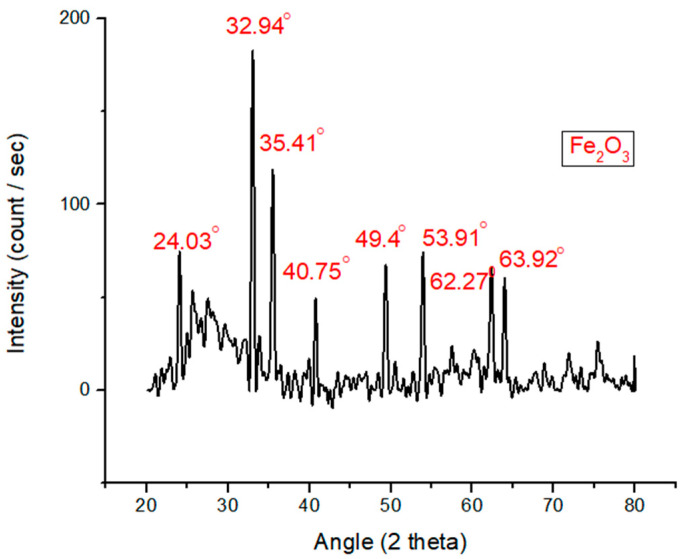
X-ray diffraction analysis of Fe_2_O_3_ nanoparticles.

X-ray diffraction examination of Fe_2_O_3_ nanoparticles was studied as shown in Figure 4. It was recorded that Fe_2_O_3_ nanoparticles indicate peaks at 24.03°, 32.94°, 35.41°, 40.75°, 49.4°, 53.91°, 62.27°, and 63.92°. The peak at 2θ is identical for 32.94°and 35.41°. This is attained when Fe_2_O_3_ nanoparticles are the highest and it can be equated with the peaks at 24.03°, 40.75°, 49.4°, 53.91°, 62.27°, and 63.92°.

### 2.7. Differential Scanning Calorimetry (DSC)

An instrument (Mettler Toledo thermogravmetric analysis/differential scanning calorimetry TGA/DSC-1-star system, Columbus, OH, USA) was used for differential scanning calorimetry examination. The measurement was proved to between 0 °C and 400 °C with a hotness expanding degree of 10 °C/min in a Nitrogen stream of 10 mL/min.

### 2.8. Thermo-Gravimetric Analysis (*TGA*) 

Thermo-gravimetric investigation was accompanied using (Mettler Toledo thermogravmetric analysis/differential scanning calorimetry TGA/DSC-1-star system, Columbus, OH, USA) device. The extent was conceded between 0 °C and 600 °C with a heat expanding proportion of 10 °C/min in a N_2_ stream of 10 mL/min.

### 2.9. Analysis of Variance (ANOVA) Nano-Composite

A one-way Analysis of Variance (ANOVA) was carried out for statistical investigation with origin 9, 32-bit software (OriginLab, Northampton, MA, USA).

## 3. Results and Discussion

Based on the previous work reported in [14,17,21], it was found that the physical performance of the wood fiber composites was low for silver, zinc and reinforced nanoparticles. In addition, the presence of these nanoparticles in MDF composites resulted reduction in the physical performance. Therefore, this research work focused on the physical properties of urea formaldehyde resin reinforced with poplar wood fibers. The effect of iron oxide nanoparticles and structural and thermal characterization of UF-Iron oxide nanofillers were studied. The influence of three different iron oxide loadings, i.e., 0.5, 1.5 and 2.5 wt %, on the physical properties of MDF composites were analyzed and are discussed in the following subsections (Section 3.1, Section 3.2, Section 3.3, Section 3.4 and Section 3.5).

### 3.1. Cured UF-Fe_2_O_3_ Nano Fluids SEM

**Figure 5:**
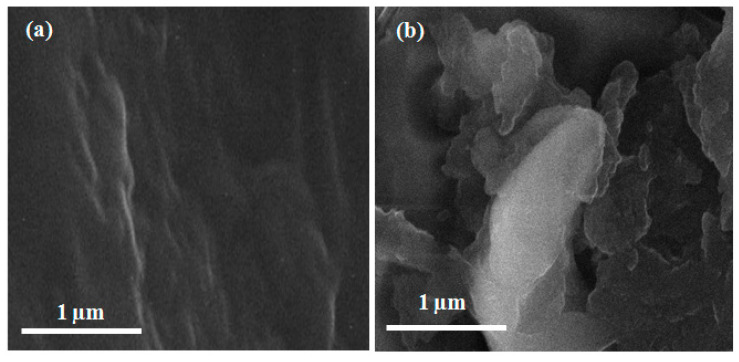
Scanning electron microscopy images of (**a**) Pure UF glue (**b**) Fe_2_O_3_-UF glue.

UF-Fe_2_O_3_ nanofluids were analyzed for surface morphology and structural analysis and can be seen in Figure 5. An odd structure of linkages of the resin was observed and visible fractional ditches were examined. These ditches were enclosed by 2.5% Fe_2_O_3_ nanoparticles concentration in urea-formaldehyde resin. The strength of the final composite becomes stronger due to the coverage of unwanted cracks and gapes by Fe_2_O_3_ nanoparticles [16]. The bright area in the scanning electron microscopy demonstrates the manifestation of Fe_2_O_3_ nanoparticles and the black area represents UF glue. The consequence was confirmed with Energy Dispersive X-ray Spectroscopy (EDS).

### 3.2. Energy Dispersive X-ray Spectroscopy (EDS) Analysis

**Figure 6:**
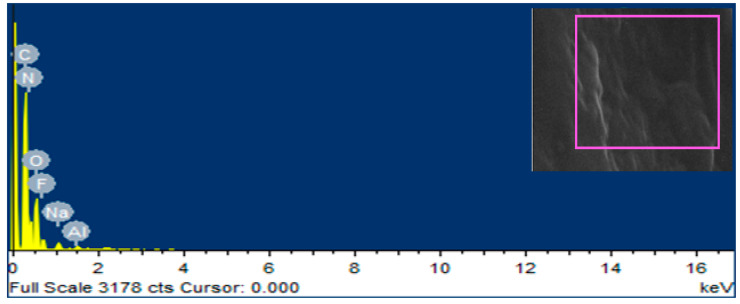
Energy dispersive spectroscopy (EDS) analysis of UF glue.

**Figure 7:**
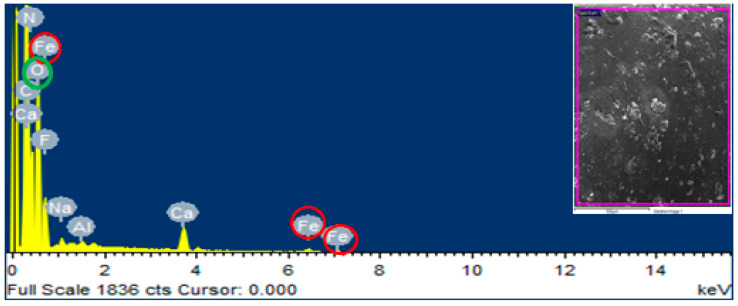
EDS of UF glue containing 5 g Fe_2_O_3_ nanoparticles.

EDS was conceded to provethe existence of Fe_2_O_3_ nanoparticles in the UF glue observed in SEM analysis. The mapping area for EDS analysis is shown in Figure 6 and Figure 7. One sample with 0% Fe_2_O_3_-UF resin and another with 2.5% Fe_2_O_3_-UF resin were designated for energy dispersive X-ray spectroscopy examination. In the subject analysis, for 0% Fe_2_O_3_ nanoparticles containing resin, no energy peak of iron was observed, while in the case of 2.5% Fe_2_O_3_ nanoparticles-based resin, three energy peaks of iron at various locations were observed. Energy peaks matched to iron and oxygen features in the tester were investigated more than the UF glue.

### 3.3. Differential Scan Calorimetry (*DSC*) of Urea-Formaldehydewith and without Fe_2_O_3_ Nanoparticles

**Figure 8:**
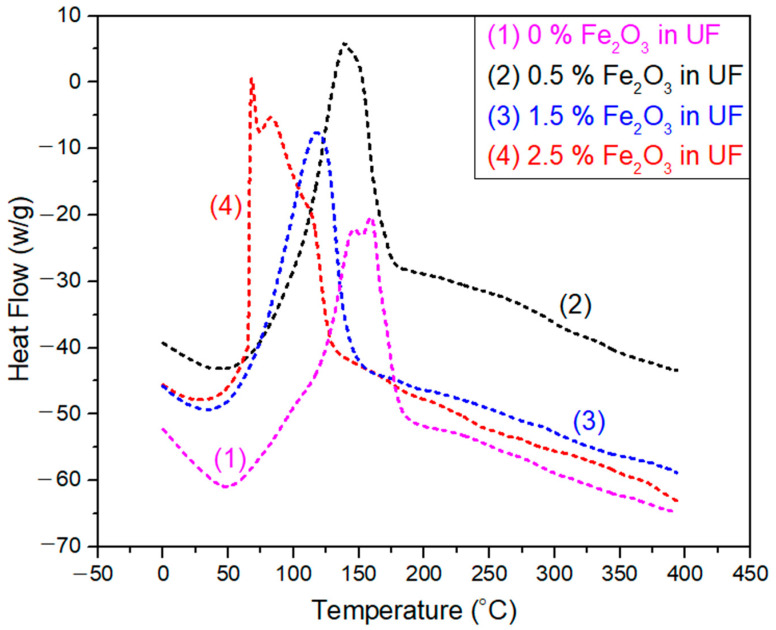
Differential scanning calorimetry (DSC) of Fe_2_O_3_-UF nanofluids.

The DSC analysis was carried out for 0, 0.5%, 1.5, and 2.5% Fe_2_O_3_ nanoparticles concentration levels as depicted in Figure 8. A demonstration of the relationship between heat flow and temperature is presented for all samples. An inverse relation between curing temperature and Fe_2_O_3_ nanoparticles concentration was observed. As the concentration of nanoparticles increase, the drying temperature falls while the amount of total heat content rises linearly with Fe_2_O_3_ nanoparticles concentration. The peak at 125 °C in 1.5% Fe_2_O_3_ nanoparticles is obtained due additional bonding formed in the UF resin. The same effect had already been shown by other thermosetting resin, as reported by Kumar, A et al. [31]. From this study it is also investigated whether early curing of the resin occurs because of Fe_2_O_3_ nanoparticles. These particles speed up the polymerization process inside the urea-formaldehyde resin and ultimately increase the heat transfer rate.

### 3.4. TGA Analysis of UF Resin with and without Fe_2_O_3_ Nanoparticles

**Figure 9:**
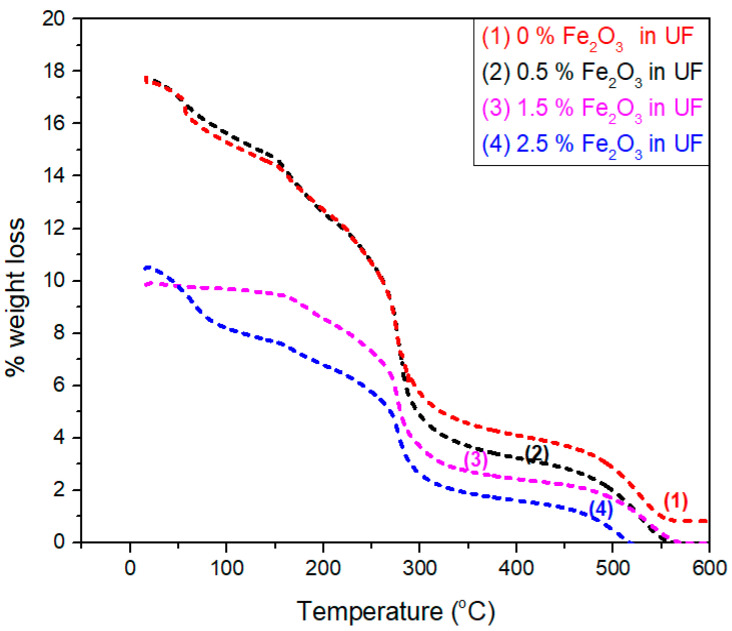
Thermogravimetric analysis (TGA) analysis of Fe_2_O_3_-UF nanofluids.

The relationship between weight loss and temperature are described in TGA curves for selected four samples, i.e., 0, 0.5, 1.5, and 2.5% Fe_2_O_3_ nanoparticles-based urea-formaldehyde resin, as shown in Figure 9. Moisture absorbed and dehydration of the resin lead to a small variation in weight loses with a temperature array of 50−150 °C, as investigated by Alabduljabbar et al. [32]. Substantial weight loss has been perceived owing to the humiliation of UF glue. The reason behind this statement is the existence of carbon (C) and hydrogen (H) bonds with inter and intramolecular interaction. Another conclusion can also be drawn from the statement that urea-formaldehyde resin contains a nitrogen (N) bond in an arbitrary division of the line. Urea-formaldehyde resin contains functional groups such as amide (C=O), (C–N), hydroxyl, and amine. These functional groups further hydrolyzed and lead to increased moisture content, unlike urea-formaldehyde containing Fe_2_O_3_ nanoparticles.

High thermal stability is achieved due to the van der Walls forces and powerful bonding exists in Fe_2_O_3_ nanoparticles-based resin [33]. The degradation occurs at temperatures from 170 °C to 480 °C.

### 3.5. Analysis of Variance (ANOVA) Nano-Composite for Physical Properties

**Figure 10:**
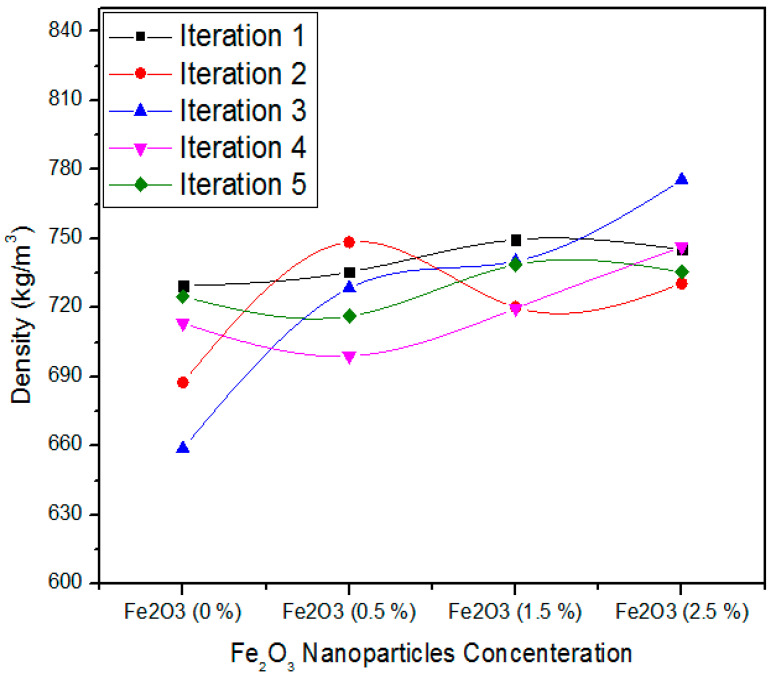
Statistical values of density of MDF for various concentration of Fe_2_O_3_ nanoparticles.

Figure 10 illustrates the one-way ANOVA consequences of assessment of five iterations of density intended for 0, 0.5, 1.0, and 2.5% absorption ranks of Fe_2_O_3_ nanoparticles. On behalf of 0% Fe_2_O_3_, the five iterations parameters of density are 729.40, 687.51, 658.80, 713.23, and 724.90 kg/m^3^. For 0.5% Fe_2_O_3_ the five iterations values of density are 735.47, 748.50, 728.45, 699.03, and 716.39 kg/m^3^. In a similar context, for 1.5% Fe_2_O_3_ nanoparticles, all the five treatments have 749.56, 720.18, 740.17, 719.70, and 738.60 kg/m^3^ density values. As the absorption level increases from 1.5 to 2.5%, the density values 745.30, 730.52, 775.50, 746.40, and 735.50 kg/m^3^ indicate substantial strengthening for all iterations.

**Figure 11:**
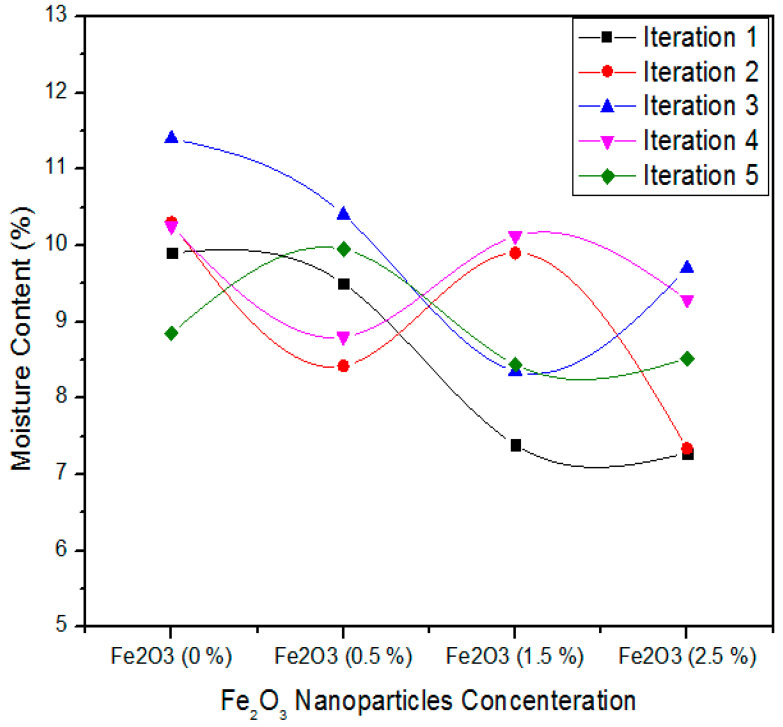
Statistical values of moisture content of MDF for various treatments of Fe_2_O_3_ nanoparticles.

Table 4 shows the detail of the one-way ANOVA of density values designed for five iterations of 0, 0.5, 1.0, and 2.5% Fe_2_O_3_ nanoparticles. The 0% Fe_2_O_3_ comprising MDF has an average density value of 702.76 kg/m^3^ and variance of 868.36. The 0.5, 1.5, and 2.5% Fe_2_O_3_ comprehending MDF have 725.56, 733.64, and 746.61 density average values with variance 355.8, 174.04, and 304.67, respectively. These density values are different from each other and the one-way ANOVA significances prove a probability (*p*-value) equal to 0.026. The moisture content property was also investigated using one-way ANOVA statistical analysis, as shown in Figure 11.

It was observed that for 0% concentration of Fe_2_O_3_ nanoparticles that the five iteration values are 9.90, 10.30, 11.40, 10.25, and 8.85%. In case of a 0.5 concentration level of Fe_2_O_3_ nanoparticles, these values were recorded as 9.50, 8.42, 10.40, 8.80, and 9.95%. Likewise, it was also analyzed that 7.39, 9.90, 8.35, 10.12, and 8.44% iteration values of moisture content exist for 1.5% Fe_2_O_3_ nanoparticles concentration in urea-formaldehyde resin. Finally, a similar approach for the highest concentration (2.5%) Fe_2_O_3_ nanoparticles was depicted as 7.28, 7.34, 9.70, 9.29, and 8.52% moisture content.

Table 5 shows the one-way ANOVA statistical technique of moisture content values for five iterations of 0, 0.5, 1.0, and 2.5% Fe_2_O_3_ nanoparticles. A level of 0% Fe_2_O_3_ comprising MDF contributes moisture content mean assessment of 10.14% and variance of 0.83. In contrast, 0.5, 1.5, and 2.5% Fe_2_O_3_ grasping MDF obtain 9.41 and 8.84 average moisture content values with variance 0.65, 1.3, and 1.21, respectively. These moisture content values are different from each other and the one-way ANOVA method proves that the possibility (*p*-value) is 0.07732.

**Figure 12:**
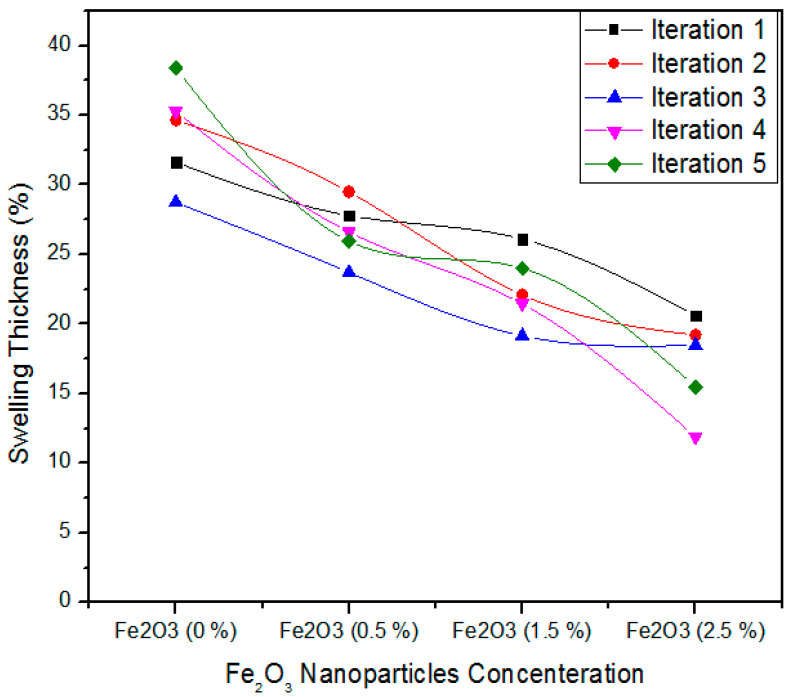
Statistical analysis of Thickness Swelling of MDF for numerous iterations of Fe_2_O_3_ nanoparticles.

Figure 12 expresses the one-way ANOVA of five iterations measurement of thickness swelling for 0, 0.5, 1.5, and 2.5% absorption levels of Fe_2_O_3_ nanoparticles. In case of 0% Fe_2_O_3_, the five iterations values of thickness swelling are 31.63, 34.64, 28.74, 35.28, and 38.37%. For 0.5% Fe_2_O_3_, the five iteration values of thickness swelling are 27.7, 29.5, 23.71, 26.62, and 25.97%. In the same way, for 1.5% Fe_2_O_3_ nanoparticles, all the five treatments have 26.1, 22.1, 19.16, 21.45, and 24% thickness swelling values. It might be possible to state that, as the absorption level increases from 1.5 to 2.5%, the thickness swelling values 201.6, 19.21, 18.48, 11.9, and 15.5% show a significant decrease for all treatments.

Table 6 presents the one-way ANOVA statistical methodology of thickness swelling values for five iterations of 0%, 0.5%, 1.5% and 2.5% Fe_2_O_3_ nanoparticles. The 0% Fe_2_O_3_ containing MDF has an average thickness swelling (Ts) value of 33.73% and alteration of 13.51. In contrast, 0.5, 1.5, and 2.5% Fe_2_O_3_ based medium density fiberboard have 26.72, 22.56, and 17.14% thickness swelling average values with variances 4.63, 6.90 and 12.06, respectively. The probability (*p*-value) is equal to 96 × 10^−6^.

**Figure 13:**
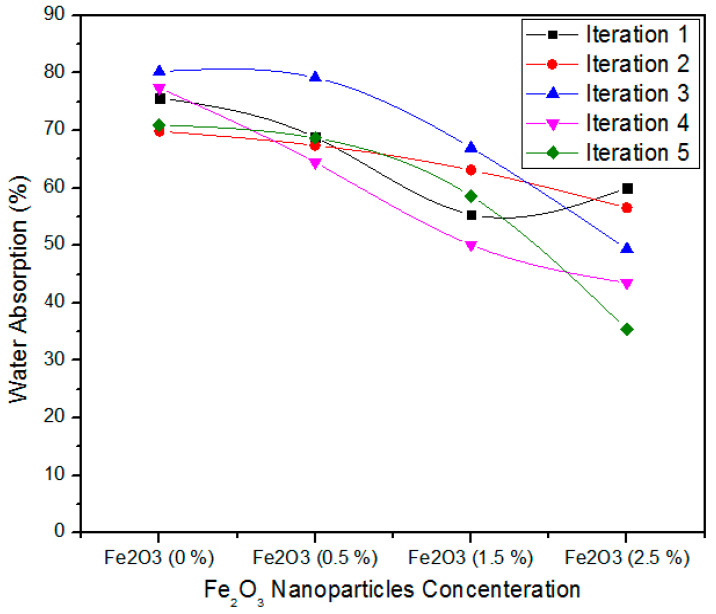
Nano-MDF water absorption parameters for numerous Fe_2_O_3_ nanoparticles concentrations.

Figure 13 illustrates the one-way ANOVA of five iterations assessment of water absorption for 0, 0.5, 1.5, and 2.5% concentration ranks of Fe_2_O_3_ nanoparticles. For 0% Fe_2_O_3_, the five iterations values of water absorption are75.65, 69.87, 80.25, 77.39, and 70.95. For 0.5% Fe_2_O_3_, the five iterations values of water absorption are 68.8, 67.43, 79.2, 64.45, and 68.7%. In contrast, for 1.5% Fe_2_O_3_ nanoparticles, all five iterations have 55.4, 63.12, 67, 50.13, and 58.6% water absorption parameters. When the absorption level escalates from 1.5 to 2.5%, the water absorption parameters 60, 56.57, 49.4, 43.5 and 35.4 show a trivial decline for all counts.

Table 7 describes the one-way ANOVA arithmetical method of water absorption values aimed at five iterations of 0, 0.5, 1.5, and 2.5 Fe_2_O_3_ nanoparticles. The 0% Fe_2_O_3_ covering MDF has a water absorption average value of 74.82% and alteration of 19.06. The 0.5, 1.0, and 2.5% Fe_2_O_3_ comprehending MDF has 69.71, 58.85 and 48.97 water absorption average values with variance 31.19, 43.16 and 98.41, 3.46. These thickness swelling values are altered and the one-way factor ANOVA concerns prove that the possibility (*p*-value) is equal to 0.000103037.

### 3.6. Nano-MDF Average Physical Properties

The physical properties of MDF samples were examined by means of 0, 0.5, 1.5, and 2.5% of Fe_2_O_3_ nanoparticles and UF glue. Each sample was examined for five repetitions and the mean value of the individual property was calculated.

The physical properties such as moisture content density, Ts, and WA are detailed in Table 8. The testers were inspected for 0, 0.5, 1.5, and 2.5% absorption stages of Fe_2_O_3_ nanoparticles with five iterations of each sample and the mean values were considered. Equally, Ts and WA investigations were accomplished for 24 h conferring to British Standard EN-3171993 and ASTM D517, separately.

The density rises by means of the escalation in absorption of nanofillers due to proliferation in the quantity of the nanofluids. A steady decline in the Ts values of the tasters for 24 h was detected. This is due to the decrease of apertures in the MDF panels. Likewise, the water absorption values correspondingly drop with the increase in meditation of nanofluids, which occurs in the case of enhanced drying of MDF panels in a hot press.

## 4. Conclusions

The effects of iron oxide nanoparticles on the physical properties of MDF composites were successfully investigated. The results indicate that the inclusion of iron oxide nanoparticles improved the thickness swelling and water absorption properties of composites. It is claimed that well-dispersed iron oxide particles within the urea formaldehyde matrix improved the gaps between the epoxy matrix and MDF composites, which then led to improvements in water absorption and thickness swelling. In addition, it is also concluded that the addition of iron oxide nanoparticle composite systems significantly improved the curing and heat transfer of urea formaldehyde resin. This may be due to the higher surface area and highly reactive properties of the nanoparticles. Hence, this resulted ina higher resistance to water when the MDF composites were immersed in water.

For future work, a suggestion can be added in lieu of a mixture tactic of Fe_2_O_3_, activated charcoal and alumina nanoparticles to get enhanced results for both the physical and mechanical characteristics of MDF.

## Figures and Tables

**Table 1:** Summary of Nano-Particles based Wood Composites.

S. No	Nano-Particles	I.B (MPa)	MOE (MPa)	MOR (MPa)	Density kg/cc	Ts (%) 24 h	WA (%) 24 h	References
1	(ATH) Aluminum trihydroxide	0.59	-	-	760	13.75	50.75	Hashim et al. (2005) [17]
2	(Nano-SiO_2_)	0.73	-	21	710	9.98		Lin Qiaojia et al. (2006) [18]
3	Nanoclay	1.0	2180	20.5	830	10	51	Yong Lei et al. (2007) [19]
4	sodium Montmorillonite /Nanoclay	1.0	3550	-	700	-	-	Hong et al. (2008) [20]
5	Nanoclay	-	2400	28	-	4.5	13	Faruk et al. (2008) [21]
6	(layered silicate)	0.67	2250	22.8	750	18.1	-	A. Ashori et al. (2009) [22]
7	Nano-silver	1.15	2450	20	-	27.70	61.90	Taghiyari et al. (2011) [23]
8	Nanoclay (MMT)	1.0	2000	9.0	700	6	10	Xian, D et al. 2013 [24]
9	Nano-Wollastonite	1.5	1200	22.5	660	15	55	Taghiyari et al. (2014) [25]
10	Nano-Reinforced	1.2	2500	13.5	-	29	93	Candan, Z et al. (2015) [26]
11	Wollastonite	-	1800	16	670	12.5	78	Taghiyari et al. 2016 [27]
12	Nanoclay	1.9	1730	15.2	800	14.5	42	N. Ismita et al. (2017) [28]
13	CaCO_3_/ PMMA	-	1260	16	700	11	55	Yipeng Chen et al. (2018) [29]
14	Zno Nanoparticles	-	-	-	680	24	-	Silva et al. (2019) [30]
15	Alumina	0.73	3381	40.54	743	5.99	13.73	Alabduljabbar, H et al. (2020) [31]

**Table 2:** Bonded glue (urea formaldehyde (UF)) specifications.

η (Viscosity, cps)	ρ (Density, grams/cm^3^)	pH	F. Formaldehyde	G.T (Gel Time, Sec)	SC (Solid Content, %)
200–320	1.24	8.5	0.71	57	55

**Table 3:** Configurations of Fe_2_O_3_-UF nanofluid.

	Composition			
Materials	Fe_0_	Fe_1_	Fe_2_	Fe_3_
UF	200	200	200	200
Fe_2_O_3_	0	1	3	5

**Table 4:** Density values of Fe_2_O_3_-UF MDF designed for a number of iterations.

	Groups	Iteration	Sum	Average	Variance	
	Fe_2_O_3_ (0%)	5	3513.84	702.76	868.63	
	Fe_2_O_3_ (0.5%)	5	3627.84	725.56	355.18	
	Fe_2_O_3_ (1.5%)	5	3668.21	733.64	174.04	
	Fe_2_O_3_ (2.5%)	5	3733.22	746.61	304.67	
**ANOVA**						
**Source of Variation**	**SS**	**df**	**MS**	**F**	***p*-value**	**F crit**
Between Groups	5095.733135	3	1698.57	3.99	0.026	3.23
Within Groups	6810.15796	16	425.63			
Total	11,905.8911	19				

**Table 5:** Moisture content values of Fe_2_O_3_-UF MDF for various iterations.

	Groups	Iteration	Sum	Average	Variance	
	Fe_2_O_3_ (0%)	5	50.7	10.14	0.83	
	Fe_2_O_3_ (0.5%)	5	47.07	9.41	0.65	
	Fe_2_O_3_ (1.5%)	5	44.2	8.84	1.31	
	Fe_2_O_3_ (2.5%)	5	42.13	8.42	1.21	
**ANOVA**						
**Source of Variation**	**SS**	**df**	**MS**	**F**	***p*-value**	**F crit**
Between Groups	8.28	3	2.76	2.74	0.07732	3.23
Within Groups	16.11	16	1.00			
Total	24.40	19				

**Table 6:** Thickness swelling values of Fe_2_O_3_-UF MDF for various iterations.

	Groups	Iteration	Sum	Average	Variance	
	Fe_2_O_3_ (0%)	5	168.66	33.73	13.51	
	Fe_2_O_3_ (0.5%)	5	133.6	26.72	4.63	
	Fe_2_O_3_ (1.5%)	5	112.81	22.56	6.90	
	Fe_2_O_3_ (2.5%)	5	85.7	17.14	12.06	
**ANOVA**						
**Source of Variation**	**SS**	**df**	**MS**	**F**	***p*-value**	**F crit**
Between Groups	734.61	03	244.87	26.38	1.96 × 10^−6^	3.23
Within Groups	148.47	16	9.27			
Total	883.09	19				

**Table 7:** Water Absorption values of Fe_2_O_3_-UF MDF for different iterations.

	Groups	Iteration	Sum	Average	Variance	
	Fe_2_O_3_ (0%)	5	374.11	74.82	19.06	
	Fe_2_O_3_ (0.5%)	5	348.58	69.71	31.19	
	Fe_2_O_3_ (1.5%)	5	294.25	58.85	43.16	
	Fe_2_O_3_ (2.5%)	5	244.87	48.97	98.41	
**ANOVA**						
**Source of Variation**	**SS**	**df**	**MS**	**F**	***p*-value**	**F crit**
Between Groups	1993.91	3	664.63	13.85	0.000103037	3.23
Within Groups	767.36	16	47.96			
Total	2761.27	19				

**Table 8:** Physical properties of 15 mm MDF samples for various absorptions of Fe_2_O_3_ nanoparticles.

MDF Specimen	Density (kg/m^3^)	TS *	WA *	Mc
S_0_Fe_0_	702.76	33.73	74.82	10.14
S_1.0_Fe_1.0_	725.56	26.72	69.71	9.41
S_1.5_Fe_1.5_	733.64	22.56	58.85	8.84
S_2.5_Fe_2.5_	746.64	17.14	48.97	8.42
Standard	720 ± 20	≤12	<45	8–9

* 24 h. Density (EN-323 standard) [34], TS (EN-317 standard) [35], WA (ASTM D570 standard) [36], Mc (EN-322) [37].
